# Assessing causality in associations between cannabis use and schizophrenia risk: a two-sample Mendelian randomization study

**DOI:** 10.1017/S0033291716003172

**Published:** 2016-12-08

**Authors:** S. H. Gage, H. J. Jones, S. Burgess, J. Bowden, G. Davey Smith, S. Zammit, M. R. Munafò

**Affiliations:** 1 MRC Integrative Epidemiology Unit (IEU), University of Bristol, Bristol, UK; 2 UK Centre for Tobacco and Alcohol Studies, School of Experimental Psychology, University of Bristol, Bristol, UK; 3 Department of Public Health and Primary Care, University of Cambridge, Cambridge, UK; 4 School of Social and Community Medicine, University of Bristol, Bristol, UK; 5 MRC Biostatistics Unit, University of Cambridge, Cambridge, UK; 6 MRC Centre for Neuropsychiatric Genetics and Genomics, Cardiff University, Cardiff, UK

**Keywords:** Cannabis, genetics, Mendelian randomization, schizophrenia

## Abstract

**Background:**

Observational associations between cannabis and schizophrenia are well documented, but ascertaining causation is more challenging. We used Mendelian randomization (MR), utilizing publicly available data as a method for ascertaining causation from observational data.

**Method:**

We performed bi-directional two-sample MR using summary-level genome-wide data from the International Cannabis Consortium (ICC) and the Psychiatric Genomics Consortium (PGC2). Single nucleotide polymorphisms (SNPs) associated with cannabis initiation (*p* < 10^−5^) and schizophrenia (*p* < 5 × 10^−8^) were combined using an inverse-variance-weighted fixed-effects approach. We also used height and education genome-wide association study data, representing negative and positive control analyses.

**Results:**

There was some evidence consistent with a causal effect of cannabis initiation on risk of schizophrenia [odds ratio (OR) 1.04 per doubling odds of cannabis initiation, 95% confidence interval (CI) 1.01–1.07, *p* = 0.019]. There was strong evidence consistent with a causal effect of schizophrenia risk on likelihood of cannabis initiation (OR 1.10 per doubling of the odds of schizophrenia, 95% CI 1.05–1.14, *p* = 2.64 × 10^−5^). Findings were as predicted for the negative control (height: OR 1.00, 95% CI 0.99–1.01, *p* = 0.90) but weaker than predicted for the positive control (years in education: OR 0.99, 95% CI 0.97–1.00, *p* = 0.066) analyses.

**Conclusions:**

Our results provide some that cannabis initiation increases the risk of schizophrenia, although the size of the causal estimate is small. We find stronger evidence that schizophrenia risk predicts cannabis initiation, possibly as genetic instruments for schizophrenia are stronger than for cannabis initiation.

## Introduction

It is clear from human experimental studies that tetrahydrocannabinol (THC) intoxication can induce transient psychotic experiences (D'Souza *et al.* 2004). However, whether chronic cannabis use is causally associated with psychotic disorders such as schizophrenia is harder to ascertain (Gage *et al.* 2013, 2016*a*). A systematic review and meta-analysis (Moore *et al.* 2007) of longitudinal studies found evidence for a modest association between cannabis and later psychotic outcomes. However, the authors noted that the studies that adjusted for more potential confounding factors found that the point estimates attenuated to a greater degree than those adjusting for fewer, indicating that residual confounding could still be present. Some studies (Callaghan *et al.* 2012; Rossler *et al.* 2012), although not all (Gage *et al.* 2014), conducted since this meta-analysis have also found evidence of an association between cannabis use and later psychotic experiences or schizophrenia. As well as these observational studies, studies have been undertaken to investigate the genetic relationships between cannabis and schizophrenia. Power *et al.* (2014) found that a genetic risk score for schizophrenia predicted cannabis use in a sample of 2082 healthy individuals. Verweij *et al.* (in press) used linkage disequilibrium (LD) score regression to investigate this association, and found evidence of a genetic correlation between lifetime cannabis use and risk of schizophrenia. This could be interpreted as shared genetic aetiology, but could also reflect a causal association between schizophrenia and risk of cannabis use (Gage *et al.* 2015).

Observational epidemiology alone cannot allow researchers to determine causality. Residual confounding and reverse causation are difficult or impossible to rule out, and associations that appear robust observationally are sometimes shown to be spurious when experimentally tested (Davey Smith & Ebrahim, 2001). A further difficulty in conducting observational studies to investigate the association between cannabis use and psychosis is that it is not possible to rule out the impact of psychotic phenomena occurring during intoxication in regular users due to the long half-life of THC (Huestis *et al.* 1992). One technique that can allow causal inference from observational data is Mendelian randomization (MR). It has been described in detail elsewhere (Davey Smith & Hemani, 2014; Gage *et al.* 2016*b*); briefly, if genetic variants can be identified that robustly predict an exposure of interest, these can be used as unconfounded proxies for that exposure. Associations between the variants and the outcome of interest can thus provide evidence of causation while (subject to certain assumptions) eliminating problems of confounding or reverse causation. A recent genome-wide association study (GWAS) of cannabis initiation (Stringer *et al.* 2016) did not find any single nucleotide polymorphisms (SNPs) that reached genome-wide significance, but identified a number of SNPs with weaker evidence of association with cannabis initiation.

Two-sample MR allows for the use of publicly available summary data, where variant-exposure associations are identified from one GWAS, and variant-outcome associations from another (Burgess *et al.* 2015*b*). This can provide the large sample sizes required to provide adequate power to identify the small effect sizes of causal effects of common genetic variants in such studies. The current study uses two-sample MR to assess the likelihood of a causal association between cannabis initiation and schizophrenia, and MR-Egger regression (Bowden *et al.* 2015) to investigate whether any association observed is due to pleiotropic effects of SNPs rather than causal effects of cannabis on schizophrenia. We also use two-sample MR with two other outcomes, years in education and height, as positive and negative controls, respectively. Evidence of association between cannabis and a positive control (where causal effects are more strongly established), and lack of evidence of association with a negative control (where causal effects are implausible) add support to causal interpretations. We chose education as a positive control as previous epidemiological evidence has suggested a probable causal association between cannabis and educational attainment (Meier *et al.* 2012). We chose height as a negative control outcome because cannabis is unlikely to have a causal effect on height since cannabis use is not common before mid- to late-adolescence (Eastwood, 2013).

## Method

The International Cannabis Consortium (ICC) GWAS, across the genome, explained 13–20% of the observed phenotypic variation of ever use of cannabis (Stringer *et al.* 2016). Although no genome-wide significant SNPs were identified, 153 SNPs were associated with cannabis initiation (ever/never use of cannabis) at *p* < 10^−5^. A number of these SNPs were in high linkage disequilibrium. Where *r*^2^ > 0.9, one SNP was randomly selected and the other highly correlated SNPs were removed from the analyses. Twenty-two SNPs remained after this pruning, and a further SNP was removed due to poor quality (Vink, 2016, personal communication). The final 21 SNPs are listed in Table 1. Correlations *r*^2^ < 0.9 were included in the analysis as a correlation matrix (Burgess *et al.* 2015*b*) (listed in Supplementary Table S1).

**Table 1.** List of SNPs (correlated < 0.9) associated with cannabis initiation (p < 10^−5^), and proxies where used

rs number	Chromosome	Nearest gene (within 100000 base pairs)	*β* (s.e.) cannabis initiation	*β* (s.e.) schizophrenia	Proxy	*r*^2^ for proxy
rs7675351	4	SCOC	0.15 (0.03)	0.00 (0.02)		
rs7700636	5	NR3C1	0.15 (0.03)	0.01 (0.01)		
rs17237367	15	RORA	0.12 (0.03)	0.01 (0.01)		
rs4984458^a^	15	–	0.10 (0.02)	−0.01 (0.01)	rs12906344	1.000
rs4984460	15	–	0.12 (0.03)	−0.01 (0.01)		
rs3738226	1	RCSD1	0.10 (0.02)	0.00 (0.01)		
rs74944517^a^	2	LOC129656	0.29 (0.06)	0.02 (0.02)	rs1526674	1.000
rs2326313^a^	3	CADM2	0.10 (0.02)	−0.01 (0.01)	rs9819830	1.000
rs6840574	4	–	0.15 (0.03)	0.01 (0.01)		
rs1554927	8	CALB1	0.10 (0.02)	0.02 (0.01)		
rs12789616	11	NCAM1	0.09 (0.02)	0.02 (0.01)		
rs8041045^a^	15	–	0.10 (0.02)	0.00 (0.01)	rs12439562	0.971
rs73067624^a^	1	KCNT2	0.18 (0.04)	0.02 (0.02)		
rs12518098^a^	5	ZSWIM6	0.10 (0.02)	0.02 (0.01)		
rs353253^a^	5	PCYOX1L	0.11 (0.03)	−0.01 (0.01)		
rs8102250^a^	19	CRX	0.13 (0.03)	0.01 (0.01)		
rs113019398^a^	20	LINC00687	0.17 (0.04)	−0.03 (0.02)		
rs2033867^a^	2	SP9	0.24 (0.05)	0.05 (0.02)		
rs13063578^a^	3	SETD2	0.11 (0.02)	0.02 (0.01)		
rs7107987^a^	11	CHID1	0.27 (0.06)	0.01 (0.01)		
rs12313672^a^	12	RNA5SP356	0.14 (0.03)	0.02 (0.01)		

a Single nucleotide polymorphisms (SNPs) not in education/height genome-wide association study.

These 21 SNPs were then extracted from the schizophrenia Psychiatric Genetics Consortium (PGC2) GWAS (Schizophrenia Working Group of the Psychiatric Genomics Consortium, 2014), a GWAS of educational attainment by the Social Science Genetics Association Consortium (SSGAC; Rietveld *et al.* 2013) and the Genetic Investigation of Anthropometric Traits (GIANT; Wood *et al.* 2014) GWAS of height. All 21 were in the schizophrenia GWAS, eight were in the education GWAS, and nine were in the height GWAS. We used the PhenoScanner tool (Staley *et al.* 2016) (http://phenoscanner.medschl.cam.ac.uk) to identify proxy SNPs using *r*^2^ > 0.9 correlations calculated in European-descent individuals from 1000 Genomes Phase 3, and a further four were identified, meaning that 12 SNPs were available for the cannabis-education and cannabis-height associations. These SNPs were used for the main analyses for the positive and negative controls. The full 21 SNPs were used for the main analysis to investigate the association between cannabis initiation and schizophrenia. We also conducted a sensitivity analysis restricting the cannabis-schizophrenia analysis to the same 12 SNPs used for the positive and negative control analyses. We used PhenoScanner to obtain estimates of the mutual correlations between variants, and a correlation matrix was included in the regression analysis model (Burgess *et al.* 2015*a*) (correlation matrix shown in Supplementary Table S1).

To assess the likelihood of a causal association between schizophrenia and cannabis initiation (schizophrenia risk predicting likelihood of cannabis initiation), we extracted 128 SNPs independently associated with schizophrenia at genome-wide significance. These SNPs explain approximately 18% of the observed variance in schizophrenia risk (Schizophrenia Working Group of the Psychiatric Genomics Consortium, 2014). Of the 128 SNPs, 107 were also present in the GWAS of cannabis initiation (listed in Supplementary Table S2).

### Effect of cannabis initiation on schizophrenia risk

Log odds ratios (ORs) and s.e. for the associations between the significant (*p* < 10^−5^) SNPs and cannabis initiation were recorded. The same SNPs were then identified in the PGC2 GWAS of schizophrenia, and the corresponding log ORs and s.e. were recorded. The SNP-exposure and SNP-outcome coefficients were combined using an inverse-variance-weighted approach to give an overall estimate of causal effect. This is equivalent to a weighted regression of the SNP-outcome coefficients on the SNP-exposure coefficients with the intercept constrained to zero.

The results of the analyses were converted to ORs. Given that schizophrenia is a binary outcome, the resulting causal effect estimate represents the odds for schizophrenia risk per unit increase in the log OR for cannabis initiation, and is therefore somewhat hard to interpret. ORs have therefore been converted (by multiplying log ORs by 0.693 and then exponentiating) in order to represent the OR per doubling in odds of the binary exposure.

### Effect of schizophrenia risk on cannabis initiation

As above, the 107 SNPs associated with schizophrenia were extracted from the GWAS schizophrenia, and then identified in the GWAS of cannabis initiation, and log ORs and s.e. recorded. The SNP-exposure and SNP-outcome coefficients were combined as described above.

### Positive and negative controls

The 12 cannabis initiation SNPs (or proxies where appropriate) were identified in GWAS of education and height, and *β* coefficients and s.e. recorded, as described above. Although these GWASs were conducted on continuous outcomes, the resulting coefficients have been transformed as described above (multiplied by 0.693) for consistency.

### Sensitivity analyses

We used MR-Egger regression to formally test for potential violations of MR assumptions. This method relaxes the assumption that the effects of genetic variants on the outcome operate entirely via the exposure, by allowing an intercept term in the weighted regression described above. The intercept parameter represents the overall pleiotropic effect of the SNPs on the outcome (i.e. a direct effect on the outcome rather than via the exposure, which would violate MR assumptions). If the intercept deviates from the null, this provides evidence that there may be bias in the standard causal estimate due to pleiotropy. The *β* coefficient for this analysis provides a causal estimate, assuming that the pleiotropic effect of SNPs on the outcome is not correlated with the associations between the SNPs and the exposure (MR-Egger is described in more detail in Corbin *et al.* 2016).

With correlated genetic variants, the inverse-variance-weighted regression model is extended to a generalized weighted regression model, as described previously (Burgess *et al.* 2015*a*). For MR-Egger regression, the same generalized weighted regression analysis was undertaken except including an intercept term in the regression model. The genetic associations with the outcome (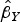) were regressed on the genetic associations with the exposure (cannabis initiation, 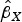) using inverse-variance weights. The regression model is:
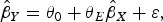
where *θ*_0_ is the intercept term and *θ*_E_ is the Egger causal estimate and *ε* is an error term with zero mean and covariance matrix Ω. The weighting matrix Ω has terms 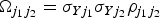, where *σ*_*Yj*_ is the s.e. of the genetic association with the outcome for the *j*th SNP, and 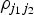 is the correlation between the *j*_1_th and *j*_2_th SNPs. This regression gives the same estimates as standard MR-Egger regression with uncorrelated variants when the correlations between different variants are all zero.

As another sensitivity analysis, the cannabis-schizophrenia association was also assessed using the 12 SNPs available for the education and height analyses, in order to allow a direct comparison. Given the potentially weak instruments used to predict cannabis initiation, we ran a further sensitivity analysis where we systematically removed each SNP from the analysis, in order to check the robustness of the findings.

Finally, we performed a calculation in order to estimate the relative risk of schizophrenia predicted by our upper confidence bound (UCB) comparing an individual who is likely to use cannabis (50% probability of initiation) with an individual with a low probability of smoking cannabis (10% probability of initiation) using the calculation: exp((log(0.5/(1–0.5)) – log(0.1/(1 – 0.1))*log(UCB/0.693).

## Results

### Effect of cannabis initiation on schizophrenia risk

In 36 989 schizophrenia cases and 113 075 controls, an MR analysis using 21 SNPs associated (*p* < 10^−5^) with cannabis initiation indicated a small association with schizophrenia case status [OR 1.04, 95% confidence interval (CI) 1.01–1.07 per doubling of the odds of initiation, *p* = 0.019].

### Effect of schizophrenia risk on cannabis initiation

In 14 388 individuals who have used cannabis in their lifetime and 17 942 non-cannabis users, an MR analysis using 107 SNPs associated with schizophrenia at genome-wide significance (*p* < 5 × 10^−8^) provided strong evidence of an association between schizophrenia case status and lifetime cannabis initiation (OR 1.10, 95% CI 1.05–1.14 per doubling of the odds of schizophrenia, *p* = 2.64 × 10^−5^).

### Positive and negative controls

In 126 599 individuals, an MR analysis using 12 SNPs associated (*p* < 10^−5^) with cannabis initiation indicated a weak association with fewer years in education (OR 0.99, 95% CI 0.97–1.00 per doubling of the odds of initiation, *p* = 0.066) (the positive control). In 253 288 individuals, an MR analysis using these SNPs found no evidence of an association with height (OR 1.00, 95% CI 0.99–1.01, *p* = 0.90) (the negative control). These results are shown in Table 2.

**Table 2:** Associations between cannabis initiation and various outcomes and schizophrenia and cannabis initiation using two-sample Mendelian randomization

Exposure	Outcome	OR^a^	95% CI	*p* value	*N* SNPs
Cannabis initiation	Schizophrenia (binary outcome)	1.04^b^	1.01–1.07	0.019	21
Cannabis initiation	Education (years)	0.99^b^	0.97–1.00	0.066	12
Cannabis initiation	Height (*z* score)	1.00^b^	0.99–1.01	0.901	12
Cannabis initiation	Schizophrenia (binary outcome)	1.03^b^	0.99–1.08	0.145	12^c^
Schizophrenia	Cannabis initiation (binary outcome)	1.10	1.05–1.14	2.64 × 10^−5^	107

OR, Odds ratio; CI, confidence interval; SNP, single nucleotide polymorphism.

a Per doubling in odds of exposure.

b Correlated likelihood method used.

c Restricted number of SNPs to be comparable to positive/negative control analyses.

### Sensitivity analyses

A sensitivity MR-Egger regression indicated very little evidence of pleiotropy in the association between cannabis and schizophrenia using the full list of 21 SNPs (intercept: OR 0.99, 95% CI 0.98–1.01, *p* = 0.50). The strength of evidence for a causal association was considerably weaker in this analysis with wider CIs (OR 1.01, 95% CI 0.93–1.10, *p* = 0.815), although not inconsistent with the findings from the primary analysis.

There was no strong evidence of pleiotropy in the association between schizophrenia (exposure) and cannabis initiation (outcome) (intercept: OR 1.00, 95% CI 0.98–1.01, *p* = 0.52). The point estimate was larger in this analysis as compared to the standard MR approach, however the strength of evidence of an association was weaker (OR 1.17, 95% CI 0.96–1.43, *p* = 0.13). The Egger plots for these analyses are shown in Supplementary Figs S1 and S2.

MR-Egger regression suggested weak evidence of pleiotropy in the positive and negative control outcome analyses using 12 SNPs (intercept OR education: 0.99, 95% CI 0.97–1.00, *p* = 0.18; height: 0.99, 95% CI 0.98–1.00, *p* = 0.093). The causal estimate from the MR-Egger regression for the association of cannabis initiation with years of education was slightly larger than for the main analysis, although the strength of evidence was very similar (OR 0.94, 95% CI 0.88–1.00, *p* = 0.062). The causal estimate from the MR-Egger regression analysis of cannabis initiation and height provided weak evidence for cannabis initiation being associated with shorter height (OR 0.95, 95% CI 0.88–1.01, *p* = 0.095), but was still consistent overall with the lack of association indicated in the primary analysis. These results are shown in Table 3.

**Table 3:** MR-Egger regression analyses showing intercept and causal estimate values for associations between cannabis initiation and various outcomes and schizophrenia and cannabis initiation

Exposure	Outcome		OR^a^	95% CI	*P*	*N* SNPs
Cannabis initiation	Schizophrenia (binary outcome)	Intercept	0.99^b^	0.98–1.01	0.50	21
		Causal estimate	1.01^b^	0.93–1.10	0.815	
Cannabis initiation	Education (years)	Intercept	0.99^b^	0.99–1.00	0.182	12
		Causal estimate	0.94^b^	0.88–1.00	0.062	
Cannabis initiation	Height (*z* score)	Intercept	0.99^b^	0.98–1.00	0.093	12
		Causal estimate	0.95^b^	0.88–1.01	0.095	
Cannabis initiation	Schizophrenia (binary outcome)	Intercept	1.00^b^	0.97–1.03	0.801	12^c^
		Causal estimate	1.01^b^	0.88–1.17	0.854	
Schizophrenia	Cannabis initiation (binary outcome)	Intercept	1.00	0.98–1.01	0.524	107
		Causal estimate	1.17	0.96–1.43	0.128	

OR, Odds ratio; CI, confidence interval; SNP, single nucleotide polymorphism.

a Per doubling in odds of exposure.

b Correlated likelihood method used.

c Restricted number of SNPs to be comparable to positive/negative control analyses.

When the association between cannabis initiation and schizophrenia risk was restricted to the 12 SNPs available for the education and height analyses, the resulting association was similar to the primary analysis, although CIs were wider (OR 1.03, 95% CI 0.99–1.08, *p* = 0.15). This is shown in Tables 2 and 3.

When we systematically removed each SNP that predicted cannabis initiation in turn there was no evidence that the size of the effect was being driven by one SNP (Supplementary Fig. S3).

Finally, we calculated the relative risk of schizophrenia for an individual who is likely to smoke cannabis (50% probability of initiation) to be at most 24% higher compared with an individual with a low probability of smoking cannabis (10% probability of initiation) based on the upper CI for the causal effect estimate.

## Discussion

Our results provide some evidence in support of the hypothesis that cannabis initiation increases the risk of schizophrenia, although the size of the causal estimate is small. Our findings therefore do not contradict the current observational literature suggesting an association between cannabis initiation and schizophrenia of a moderate size, and may suggest any true causal effect size is smaller still. Given the small effect sizes generally seen in the observational data (Moore *et al.* 2007) particularly when considering cannabis initiation phenotypes, and in these results using a different methodology, this converging evidence supports the theory that although cannabis use might be a risk factor for schizophrenia, any effect cannabis initiation has is likely to be small. For example, based on the upper bound of the 95% CI from our primary analysis, an individual who is more likely to smoke cannabis (50% probability of initiation) has at most a 24% higher relative risk of suffering from schizophrenia compared with an individual with a low probability of smoking cannabis (10% probability of initiation). However, these results are unlikely to be directly comparable to the observational studies in terms of comparing effect sizes, particularly with regard to heavier use of cannabis, or use of different strains of cannabis where ratios of cannabinoids may differ. The ratio of THC and cannabidiol (CBD) is likely to be particularly important in determining the psychotomimetic properties of cannabis (D'Souza *et al*. 2004; Iseger & Bossong, 2015), although genetic variants that index the strength of cannabis used have not been identified as yet.

We found strong evidence in support of the reverse causation hypothesis, that schizophrenia risk predicts likelihood of cannabis initiation. These findings are consistent with a recent study that showed genetic predisposition for schizophrenia was associated with increased use of cannabis (Power *et al.* 2014). The authors of that study interpreted their findings as evidence for shared genetic aetiology between cannabis use and schizophrenia; however, an alternate interpretation, supported by the more directional analyses reported here, is that schizophrenia risk increases the risk of cannabis use. Our study cannot provide information about potential mechanisms through which this could happen. Causation could be via either biological or social mechanisms. For example, people with a higher genetic risk for schizophrenia could be more predisposed to try cannabis because they experience more pleasurable or positive effects of intoxication, or because they are more likely to associate with people who use cannabis (Nordsletten *et al.* 2016). However, these explanations remain speculative, although could be tested in further research, for example using a recall-by-genotype design. The findings from the positive and negative control analyses were broadly consistent with our prediction; our results suggest that cannabis initiation is associated with fewer years in education (although with weaker evidence than predicted), while cannabis initiation does not predict height.

### Strengths and limitations

MR studies allow stronger evidence of causality than is possible from observational epidemiology, which is a strength of this study. Utilizing both MR and positive and negative control designs allows for a triangulation of approaches with different limitations (Gage *et al.* 2016*b*). A particular strength of two-sample MR is that it can provide the large sample sizes required to identify the small effect sizes that are likely when using SNPs as proxies to study complex phenotypes. However, there are a number of limitations to our study. First, none of the SNPs used to predict cannabis initiation reached genome-wide significance in the original GWAS (Stringer *et al.* 2016). As these SNPs are not strong predictors of cannabis initiation (and some may represent false positives), it is likely that our effect size estimates will be overly conservative. The weak evidence of association with our positive control could be explained by the weakness of our genetic instrument. While these SNPs did not reach genome-wide significance in the cannabis initiation GWAS, they were nevertheless associated with initiation at *p* < 10^−5^. Inferences and estimates from MR analyses may still be valid even if genetic variants are included that are not truly associated with the exposure; their inclusion is equivalent to adding noise to a genetic risk score. Including false positive variants is not ideal as it reduces power and could introduce pleiotropic variants to the analysis. However, as we do not know which variants are true associations and which are false positives, our approach makes the best possible use of the available data. We also conduct MR-Egger sensitivity analyses, which are more robust to pleiotropy.

Secondly, there is a small overlap in the cohorts included in the schizophrenia and cannabis GWAS, as both include data from the Estonia Genome Centre cohort (EGCUT). ECGUT data makes up 2.3% of the PGC2 dataset, and approximately 10% of the Cannabis Consortium dataset. The true overlap is 1500 individuals (T. Esko, 2016, personal communication). A small overlap such as this is unlikely to result in substantial bias (Burgess *et al.* 2016). Large overlap would bias the results in the direction of the observational association (and therefore could lead to an overestimation of the effect). We reran our main analyses in both directions using a version of the PGC2 data with this sample removed. Results did not change from those presented.

Thirdly, and perhaps more problematic, is that it is not clear what the cannabis initiation phenotype used in the Cannabis Consortium GWAS actually represents; participants were asked ‘Have you ever used cannabis?’, so there is no distinction between having tried cannabis once and having used it every day for many years. If there is a causal association between cannabis and schizophrenia, it is more likely that exposure intensity would be the relevant risk factor. Observationally, the association between ever use of cannabis and later psychosis is much weaker than for heavy cannabis use (Moore *et al.* 2007). Furthermore, those who choose to try cannabis (rather than become regular or heavy users) might be more impulsive or risk taking than those who never try it, and this GWAS could therefore be a measure of that or other related phenotypes, rather than cannabis initiation *per se*. Our lack of understanding of the function of the genetic variants identified in the cannabis initiation GWAS and their role in cannabis metabolism means that we do not understand the mechanism by which they impact on cannabis use.

A recent GWAS of cannabis use identified three SNPs at genome-wide significance in a sample of 14 754 participants (Sherva *et al.* 2016). However, this GWAS conflates cannabis initiation with dependence phenotypes, which would make interpretation of any association we might find challenging. Moreover, given our inability to stratify participants in the schizophrenia PGC sample by cannabis use status, we deemed it inappropriate to use these SNPs as genetic influences on cannabis dependence cannot occur unless a person has used cannabis. This analysis needs to be conducted in cannabis users separately from non-users; if the association between cannabis and schizophrenia is causal, there should be an association observed between the cannabis dependence SNPs and schizophrenia in cannabis dependent subjects, but no association observed in non-users.

A further limitation relates to the nature of schizophrenia. Since most of the population do not have schizophrenia, we are making an assumption that genetic predictors of schizophrenia also have an impact in the general population. There is evidence that psychotic symptoms exist on a continuum (Linscott & van Os, 2013), which would mean this is an appropriate assumption to make. It has also been found that genetic risk for schizophrenia predicts childhood psychopathology (Jones *et al.* 2016).

Finally, a further limitation is our choice of positive and negative controls. While weak evidence of association may be due to the use of a weak genetic instrument, as discussed above, it is also possible that our assumption of causality between cannabis and education is misplaced. Since we began our analyses, two studies have been published suggesting that the association between cannabis and education may be more strongly confounded than previously believed (Jackson *et al.* 2016; Mokrysz *et al.* 2016). With regards to height as a negative control, there is some evidence of a negative correlation between height and schizophrenia risk that could be due to pleiotropy (Bacanu *et al.* 2013). Identifying appropriate positive and negative controls is extremely challenging when investigating complex phenotypes such as substance use and mental health. However, in the interests of completeness we present both our positive and negative control analyses as originally conceived.

Given that schizophrenia and cannabis initiation are both complex phenotypes, likely to be influenced by multiple genetic and environmental risk factors, a consideration of pleiotropy is important. We conducted MR-Egger analyses to formally test for biological pleiotropy (where genetic variants have a direct impact on more than one phenotype). Biological pleiotropy violates the assumptions of MR, but we find only weak evidence of biological pleiotropy. It is distinct from mediated pleiotropy (whereby a genetic variant may have a mediated impact on a later phenotype via a direct effect on an upstream phenotype), which does not violate the assumptions of MR. A recent paper (Verweij *et al.* in press) used LD score regression to assess the association between cannabis use and schizophrenia, and found evidence of a genetic correlation. This could mean shared genetic architecture, but it is also consistent with a causal relationship in one or both directions.

## Conclusion

Our results provide some evidence that cannabis initiation might be causally associated with odds of developing schizophrenia, using two-sample MR to support stronger causal inference, although the size of the association is small. The results provide stronger evidence that schizophrenia risk predicts likelihood of cannabis initiation, but the weakness of the currently available genetic instrument for cannabis use limits our interpretation of these results. While stronger instruments are likely to be identified with increasing GWAS sample sizes, future GWAS studies of schizophrenia should prioritise recording cumulative cannabis exposure in cases and controls to allow the causal association between heaviness of cannabis use and schizophrenia to be investigated in stratified samples.

## Supplementary material

The supplementary material for this article can be found at https://doi.org/10.1017/S0033291716003172.
